# Tracking SARS-CoV-2 in Sewage: Evidence of Changes in Virus Variant Predominance during COVID-19 Pandemic

**DOI:** 10.3390/v12101144

**Published:** 2020-10-09

**Authors:** Javier Martin, Dimitra Klapsa, Thomas Wilton, Maria Zambon, Emma Bentley, Erika Bujaki, Martin Fritzsche, Ryan Mate, Manasi Majumdar

**Affiliations:** 1 Division of Virology, National Institute for Biological Standards and Control (NIBSC), Potters Bar, Hertfordshire EN6 3QG, UK; dimitra.klapsa@nibsc.org (D.K.); thomas.wilton@nibsc.org (T.W.); emma.bentley@nibsc.org (E.B.); erika.bujaki@nibsc.org (E.B.); manasi.majumdar@nibsc.org (M.M.); 2 Respiratory Virology & Polio Reference Service, Public Health England, London NW9 5EQ, UK; maria.zambon@phe.gov.uk; 3 Division of Analytical and Biological Sciences, NIBSC, Potters Bar, Hertfordshire EN6 3QG, UK; martin.fritzsche@nibsc.org (M.F.); ryan.mate@nibsc.org (R.M.)

**Keywords:** COVID-19, wastewater, SARS-CoV-2 RNA, next-generation sequencing, variant G614, virus evolution, alter surveillance system

## Abstract

Severe Acute Respiratory Syndrome Coronavirus 2 (SARS-CoV-2), responsible for the ongoing coronavirus disease (COVID-19) pandemic, is frequently shed in faeces during infection, and viral RNA has recently been detected in sewage in some countries. We have investigated the presence of SARS-CoV-2 RNA in wastewater samples from South-East England between 14th January and 12th May 2020. A novel nested RT-PCR approach targeting five different regions of the viral genome improved the sensitivity of RT-qPCR assays and generated nucleotide sequences at sites with known sequence polymorphisms among SARS-CoV-2 isolates. We were able to detect co-circulating virus variants, some specifically prevalent in England, and to identify changes in viral RNA sequences with time consistent with the recently reported increasing global dominance of Spike protein G614 pandemic variant. Low levels of viral RNA were detected in a sample from 11th February, 3 days before the first case was reported in the sewage plant catchment area. SARS-CoV-2 RNA concentration increased in March and April, and a sharp reduction was observed in May, showing the effects of lockdown measures. We conclude that viral RNA sequences found in sewage closely resemble those from clinical samples and that environmental surveillance can be used to monitor SARS-CoV-2 transmission, tracing virus variants and detecting virus importations.

## 1. Introduction

A global pandemic of coronavirus disease (COVID-19) caused by a new betacoronavirus named Severe Acute Respiratory Syndrome Coronavirus 2 (SARS-CoV-2) is currently ongoing [1]. The outbreak was first detected in Wuhan (China) in December 2019 and spread rapidly to 213 countries/territories with 33.50 million confirmed cases and 1,004,421 deaths as of 30th September 2020 [2]. While the majority of infections result in no apparent symptoms or mild ones, some progress to acute respiratory disease, multi-organ failure, and death [3]. Respiratory transmission is the primary route for SARS-CoV-2 infection although faecal–oral transmission is possible as high levels of viral RNA have been detected in stool samples of a proportion of infected individuals [4]. Studies have shown that viral RNA of titres up to 8.0 Log10 genome copies (gc)/gram of faeces can be detected in stools of infected people for prolonged periods of time, long after the virus is no longer detected in respiratory samples [5,6,7,8]. Correspondingly, SARS-CoV-2 RNA has been found in wastewater samples in some countries with viral RNA loads ranging between 2.0 and 6.0 Log10 gc/L of sewage and generally a good correlation between RNA levels and numbers of COVID-19 confirmed cases [9,10,11,12,13,14,15,16,17,18].

SARS-CoV-2 has spread very rapidly in the population resulting in a high number of people requiring hospitalization. Consequently, many countries have been forced to implement severe lockdown measures to ensure physical distance between people and interrupt virus transmission [19,20,21,22]. These measures have largely reduced the number of confirmed cases in several countries, but outbreaks are emerging in some areas following the ease of lockdown measures [23]. As a large proportion of the population appears to remain susceptible for SARS-CoV-2 infection [24,25,26,27], the risk of new waves of infection is high. Four successive waves were observed during the global 1918 influenza pandemic, which lasted from February 1918 to April 1920 infecting an estimated 500 million people and causing between 17 and 50 million deaths [28]. As with the influenza pandemic, the quality and length of lockdown measures will determine its effectiveness in reducing deaths and future peaks of COVID-19 disease [29]. In this context, monitoring virus spread and understanding virus transmission patterns becomes critical. In a situation in which a high proportion of asymptomatic infected individuals occurs, with potential to silently spread the disease, environmental surveillance (ES) could prove to be a very useful tool to track virus circulation. A good example is poliovirus as ES has been shown to be a very sensitive method to detect poliovirus circulation [30], even in the absence of reported paralytic polio cases [31], and has helped tracing the elimination of wild and vaccine poliovirus in some areas largely contributing to global polio eradication efforts. Although there is some evidence of the early detection of viral RNA in sewage even before COVID-19 cases had been reported [10,11,32], it still remains to be determined how well virus found in sewage represents virus circulating in humans and whether ES can help in the early detection of peaks in virus transmission for a public response to be timely effective.

Apart from establishing an alert system for virus detection by ES, phylogenetic analysis of viral RNA found in sewage [14,16] can also help in understanding virus transmission patterns, tracing virus variants, and detecting virus importations. Analysis of SARS-CoV-2 RNA nucleotide sequences from clinical samples reveals some level of genetic variation including non-synonymous changes observed during the COVID-19 pandemic [33,34,35,36,37,38,39,40,41]. This includes a relevant virus variant containing an amino acid change from aspartate (D) to glycine (G) at residue 614 in the SARS-CoV-2 spike (S) protein, responsible for virus attachment to the Angiotensin-converting enzyme 2 (ACE2) receptor on host cells and subsequent cell entry. This virus, named the G614 variant, has become the dominant pandemic form globally although its biological significance is still not clear [42].

With the above scientific information in mind, we aimed at investigating whether we could detect the presence of SARS-CoV-2 in wastewater samples from England (United Kingdom) using standard real-time quantitative reverse transcription-polymerase chain reaction (RTqPCR) assays as reported in different countries. Raw inlet sewage samples collected between 14th January and 12th May 2020 were analysed. In addition, nested reverse transcription-polymerase chain reaction (nPCR) assays specifically targeting SARS-CoV-2 sequences were designed to complement the RTqPCR assays and provide nucleotide sequence information to confirm the association between viral sequences found in clinical samples and those found in sewage. This could eventually help to improve the predicting value of ES for SARS-CoV-2 detection. We have also used next-generation sequencing analysis to investigate whether sequencing data from viral RNA found in sewage can help identify changes in the predominance of different genetic variants that have been reported to occur during the current COVID-19 pandemic. Given the global relevance of the G614 pandemic variant described above, we have targeted genomic regions containing nucleotide variations found in this variant. In addition, nucleotide variants specifically prevalent in England during the period of study were also the target of our sequence analyses.

## 2. Materials and Methods

### 2.1. Wastewater Sample Collection and Processing

One litre of inlet wastewater composite samples was collected during a 24 h period at a sewage plant in South East England with a catchment area of approximately 4.0 × 10^6^ people. The samples were transported to the laboratory in chilled packages on the day of sampling and were stored at −80 °C until use. Each sample was processed using a filtration–centrifugation method described before and previously validated for the detection of polio and non-polio enteroviruses during routine ES for poliovirus as part of our role as a WHO Global Specialized polio network laboratory [43,44]. Briefly, following the removal of solids by centrifugation at 3000× *g*, wastewater was filtered through a 500 mL Nalgene Rapid-flow™ 0.45 µM filter (Thermo Fisher Scientific, Waltham, MA, USA) and concentrated using Centriprep centrifugal filter units with a 10 kDa molecular weight cutoff (Merck Life Science UK Limited, Gillingham, UK) following manufacturer’s instructions. Starting volumes of raw sewage between 120 and 240 mL yielded 4–6 mL of concentrate. These were further concentrated in a second step, when necessary, with final volumes of concentrates reaching between 150 and 350 µL. A total of five sewage samples was processed, one from each month. At least two aliquots from each raw sewage sample were processed and analysed independently. As shown in the literature, and from our own experience, we know that human viruses in sewage often show a non-homogeneous distribution, particularly when virus concentrations are low [42,45]. Aliquot samples from the same wastewater concentrate preparation almost always yield a different spectrum of polio and non-polio enterovirus serotypes when analysed by infection in cell cultures or direct molecular assays [42,45]. For this reason, following concentration of raw sewage, we tested a minimum of five replicate RNA samples from at least two independent wastewater concentration processes for each sample.

### 2.2. Quantification of SARS-CoV-2 RNA in Wastewater Concentrates by RT-qPCR

The SARS-CoV-2 RNA content in wastewater concentrates was estimated by real-time RT-qPCR using a qScript XLT qPCR Toughmix system (Quantabio, Beverly, MA, USA) in a Rotor-Gene Q instrument (Qiagen) and following a one- RT-PCR protocol. Viral RNA was purified from wastewater concentrates using the High Pure viral RNA kit (Roche Life Science, Mannheim, Germany). Previously reported primer reactions *RdRP* and *E-Sarbeco* [46] were used with the following amplification conditions: RT reaction was conducted at 50 °C for 30 min followed by 40 PCR amplification cycles of 95 °C for 15 s, 50 °C for 45 s, 61 °C for 20 s, and 72 °C for 5 s. A standard curve for SARS-CoV-2 RNA quantification was generated using serial dilutions of RNA extracted from the National Institute for Biological Standards and Control (NIBSC) virus reagent 19/304 containing noninfectious synthetic SARS-CoV-2 RNA packaged within a lentiviral vector that had been calibrated with plasmid DNA constructs to contain a concentration of 7.0 Log10 SARS-CoV-2 genome copies (gc)/mL (https://www.nibsc.org/products/brm_product_catalogue/detail_page.aspx?catid=19/304, accessed on 4 July 2020). The results were expressed in Log10 SARS-CoV-2 gc/L of sewage. Replicate assays were performed for each sample to improve quantification estimates. Good laboratory practices were observed in all assays to reduce the possibility of cross-contamination: i.e., using different laboratory locations for sample processing, preparation of reaction mixtures, template addition, and post-processing analysis. Two different operators tested each sample at least once. RNA extraction and no template controls were included in every assay and were always found to be negative. An additional RT-PCR reaction using enterovirus primers was used as process control to rule out the presence of PCR inhibitors. The presence of live human enteroviruses in wastewater concentrates was also assessed using standard cell culture procedures as part of our routine process for poliovirus surveillance [47].

### 2.3. SARS-CoV-2 Whole-Genome Sequences Used for Nucleotide Sequence Analyses

Whole-genome SARS-CoV-2 sequences collected up to 31st May 2020 were downloaded from the Global Initiative on Sharing All Influenza Data (GISAID) database [48] on 4th July 2020. Only sequences >29,000 nt in length were used in our analysis. Remarkably, 33.04% of the whole-genome SARS-CoV-2 sequences analysed from the GISAID database (18,082 of 56,899) were from England. Tables S3–S9 show acknowledgments for authors who submitted the sequences analysed.

### 2.4. Nested RT-PCR (nPCR) Amplification

Whole-genome SARS-CoV-2 viral RNA sequences were downloaded from the GISAID database [48] to identify suitable genetic markers to be used in our sequence analyses, specifically we looked at sequence variations observed between viral RNA sequences from England. Geneious R10 software (Biomatters, Auckland, New Zealand) was used for all nucleotide sequence analyses. Whole-genome sequences were aligned to a reference sequence (Wuhan-Hu-1 strain) with National Center for Biotechnology Information (NCBI) accession no. MN908947 and the frequency of sequence variation at each nucleotide position was determined by standard single nucleotide polymorphism (SNP) analysis using Geneious R10 software default settings. RT-PCR fragments corresponding to different regions across the SARS-CoV-2 genome were amplified from purified viral RNAs by one-step RT-PCR using a Invitrogen SuperScript III One-Step RT-PCR System with Platinum Taq High-Fidelity DNA Polymerase. Genome location and nucleotide sequences of primer sets used for the PCR reactions are shown in Figure S1, nPCR reactions were named nPCR1 to nPCR5. Amplification conditions were: 50 °C for 30 min followed by 94 °C for 2 min plus 40 cycles of 94 °C for 15 s, 55 °C for 30 s and 68 °C for 8 min with a final extension step of 68 °C for 5 min. Following the first PCR reaction, 1 µL of amplified product was used for the second PCR reaction using the DreamTaq™ Hot Start PCR Master Mix with the same amplification conditions used for the first PCR step. Final amplified products were purified using QIAquick^®^ PCR Purification kit (Qiagen, Manchester, UK) ready for Sanger and next-generation sequencing (NGS) analysis. Primers were tested using serial dilutions of purified RNA from NIBSC’s virus reagent 19/304 referred above. RNA extraction and no template controls were included in every assay and were always found to be negative. Primers used in this study did not closely match viral RNA sequences from seasonal coronavirus that had been circulating worldwide the last several years. Besides this, published nucleotide sequences of seasonal coronavirus serotypes in the PCR regions amplified, are at least 30% different to those from SARS-CoV-2 isolates, which means that full-sequence analysis can unequivocally demonstrate that the sequenced nPCR products from this study were from SARS-CoV-2 and not seasonal coronavirus. All nucleotide sequences of nPCR products in this study were identical or nearly identical to sequences from COVID-19 isolates from England and none resembled those from seasonal coronaviruses.

### 2.5. Sanger Sequencing Analysis of RT-PCR Products

Purified DNA products were sequenced using an Applied Biosystems Prism 3130 genetic analyser. SARS-CoV-2 sequences obtained in this study were compared to those available in the GISAID database [48]. Geneious R10 software was used for these analyses. Sanger nucleotide sequences generated for this paper are available from the GISAID database [49] with Accession IDs EPI_ISL_499042 and EPI_ISL_500801-EPI_ISL_500830.

### 2.6. NGS Analysis of RT-PCR Products

NGS libraries were constructed using the DNA Prep kit (formerly known as Nextera DNA Flex) and dual-indexed using Nextera DNA CD Indexes (both Illumina, San Diego, CA, USA). These libraries were pooled in equimolar concentrations and sequenced with 250 bp paired-end reads on MiSeq v2 (500 cycles) kits (Illumina). Initial demultiplexing was performed on-board by the MiSeq Reporter software. FASTQ sequencing data was adapter and quality trimmed by Cutadapt v2.10 [50] for a minimum Phred score of Q30, minimal read length of 75 bp, and 0 ambiguous nucleotides. Relevant FASTQ files used in this study are available from the NCBI Short Read Archive under BioProject ID: PRJNA666219.

### 2.7. Generation of SARS-CoV-2 Sequence Contigs and Identification of SNPs

Further processing and analysis of NGS data was performed with Geneious R10 software using methods described before [44,51]. Filtered reads were imported into Geneious R10, paired-end reads combined and sequence contigs built by reference-guided assembly. Reads were mapped to references with a minimum 50 base overlap, minimum overlap identity of 90%, maximum 10% mismatches per read, allowing up to 15% gaps, and index word length of 12. SNPs were identified using Geneious R10 default settings. Variants with strand bias >90%, coverage <100, average quality <30, variant frequency <5%, and the number of total variant reads <10 were excluded. RNA extracted from NIBSC’s virus reagent 19/304 was used as control to measure background sequencing error.

## 3. Results

### 3.1. Detection of SARS-CoV-2 RNA in Wastewater Samples

Following concentration of raw sewage as described in Section 2.1, we tested a minimum of five replicate RNA samples from at least two independent wastewater concentration processes for each sample. Further replicate RNAs were tested for positive samples to obtain more accurate viral RNA quantification. SARS-CoV-2 RNA in wastewater samples was quantified using a real-time quantitative polymerase chain reaction (RTqPCR) assay targeting the RNA-dependent RNA Polymerase (RdRP) gene. A second RTqPCR assay targeting the envelope protein (E) gene was used for confirmation. The *E-gene* RTqPCR assay was less sensitive and accurate as the limit of quantitation (LOQ) was higher. The LOQ was 32 genome copies of SARS-CoV-2 RNA per reaction for the *RdRP-gene* assay and 160 genome copies per reaction for the *E-gene* assay as found using RNA extracted from the NIBSC virus reagent 19/304. These LOQ values correspond to 3.50 and 4.20 Log10 gc/L of sewage, respectively, when maximum concentration is achieved. As shown in Table 1, positive RTqPCR signals were obtained for the samples from March, April, and May.

The sample from May was only positive in 3 out of 11 replicate assays with the *RdRP-gene* reaction and in none of the reactions with *E-gene* primers, so accurate quantification of viral RNA in this sample was not possible. However, it was clear that there was a large reduction of SARS-CoV-2 RNA concentration in sewage between 14th April and 12th May. Positive and negative results were independently confirmed using a second real-time PCR platform (Stratagene 3000P) in a different NIBSC laboratory. Integrity of process was confirmed through use of previous experience with enteroviruses. This was demonstrated both by detection of enteroviral RNA and recovery of infectious virus in cell cultures from all wastewater concentrates following WHO-recommended protocols as described in Section 2.

### 3.2. Analysis of Nucleotide Sequence Variation among SARS-CoV-2 RNA Sequences from Clinical Samples

**Figure 1:**
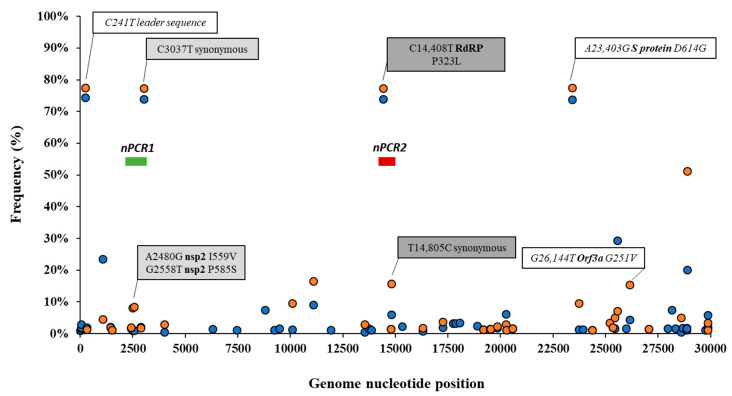
Analysis of nucleotide sequence variation among SARS-CoV-2 RNA sequences from clinical samples. Nucleotide positions at which sequence variation in >1% of viral RNA sequences from England (orange circles) or the rest of the world (blue circles) was observed are shown. Relevant nucleotide positions contained in nPCR1 (light grey shaded box) and nPCR2 (dark grey box) products are shown together with associated nucleotide variations (white boxes) observing very similar frequencies as expected. The genome locations of nPCR1 and nPCR2 products are shown in green and red boxes. The Wuhan-Hu-1 strain (NCBI accession no. MN908947) was used as reference. Whole-genome SARS-CoV-2 sequences used in this analysis were downloaded from the GISAID database [48].

Details of the number of sequences analysed by date and country are given in Table S1. The frequency of sequence variation at each genomic nucleotide position was determined with respect to the reference Wuhan-Hu-1 strain (NCBI accession no. MN908947) for each dataset. Figure 1 shows nucleotide positions at which sequence variation in >1% of viral RNA sequences from England and the rest of the world were observed.

**Figure 2:**
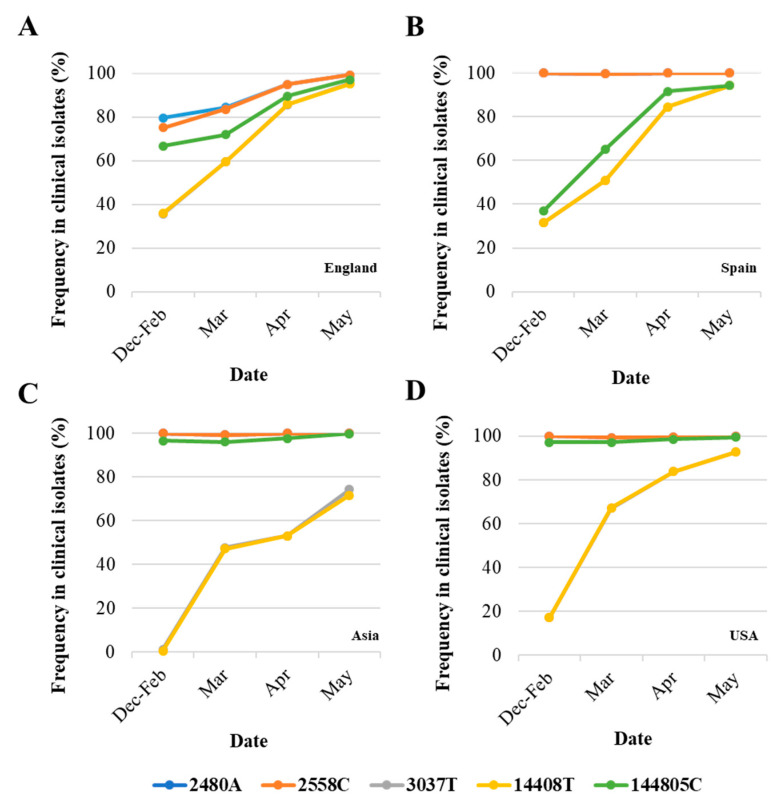
Changes in nucleotide sequence frequency at five selected SARS-CoV-2 genomic positions during the coronavirus disease (COVID-19) pandemic. The frequencies of 2480A (blue), 2558C (orange), 3037T (grey), 14408T (yellow), and 14805C (green) in England (**A**), Spain (**B**), Asia (**C**), and USA (**D**) are shown. Lines for nucleotides 2480 and 2554 and those for nucleotides 3037 and 14,408 mostly overlap as they correspond to associated sequence variations. Whole-genome SARS-CoV-2 sequences used in this analysis were downloaded from the GISAID database [48].

Differences in nucleotide sequence frequencies at some of these common positions were noticeable indicating a different prevalence of some sequence variants between viral sequences in England and the rest of the world. We used this information to select genomic regions for our sequence analysis. Key nucleotide positions 2480, 2558, 3037, 14,408, and 14,805 were targeted in two nPCR products, nPCR1 and nPCR2. nPCR1 spans nucleotides 2344–3118 and nPCR2 covers nucleotides 14,342–14,913 (Figure S1). The nPCR1 product includes nucleotide variants A2480G and C2558T, which result in amino acid changes I559V and P585S in nsp2 protein and which are often associated between them. The nPCR1 product also includes nucleotide C3037T, which is almost always associated with nucleotide sequence variations C241T, C14408T, and A23403G; mapping in the leader sequence; RNA polymerase (P323L amino acid change); and Spike protein (D614G amino acid change), respectively. This virus variant containing these four nucleotide variations, named G614, has become the dominant pandemic virus around the world [42]. The nPCR2 product includes nucleotide variant C14408T, also part of the dominant G614 pandemic strain, and synonymous change T14805C often associated with variation G26144T, which results in amino acid change G251V in Orf3a protein. The frequency of T14805C is 15.8% in England versus 6.1 % in the rest of the world. Table S2 shows how nucleotide sequences at these selected five nucleotide positions most commonly combine in SARS-CoV-2 isolates. We also show in Figure 2 how the frequency of sequence variants at these five positions has changed during the pandemic in different countries/regions of the world.

As can be noted, differences in sequence composition at these positions were notable between clinical samples from different countries/regions, likely reflecting differences in the circulation of different virus variants. Variants A2480G and C2558T were present in very low proportion in Spain, Asia, and USA as compared to the proportion in England. Variant C14480T was particularly prevalent in Spain and increase in the proportion of nucleotide variations characteristic of G614 pandemic variant was delayed in Asia with respect to the other regions analysed. Three additional nPCR assays were designed as described in Section 2 and Section 3.3 below.

### 3.3. Generation of nPCR Products for Nucleotide Sequence Analyses

**Figure 3:**
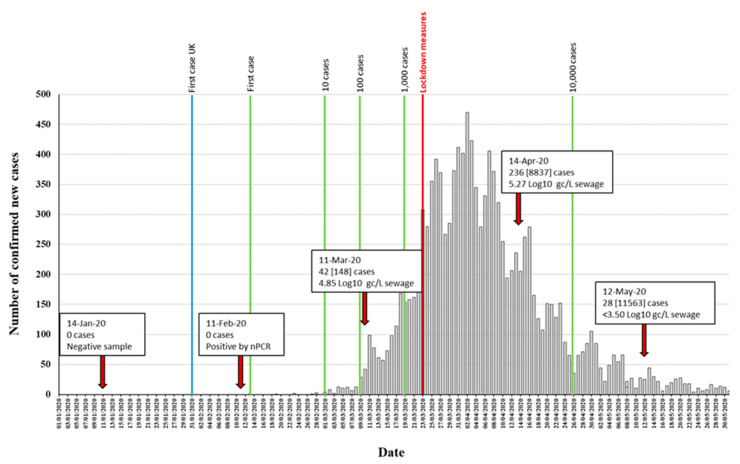
Detection of SARS-CoV-2 RNA in relation to COVID-19 confirmed cases. The number of daily reported new COVID-19 cases in the catchment area covered by the sewage plant is shown as grey columns. The time points at which 1, 10, 100, 1000, and 10,000 confirmed cases where reached in the area are indicated with green vertical lines. The blue vertical line indicates the time point of the first U.K. confirmed case, outside the sampling area. Environmental surveillance (ES) sampling time points are shown with red arrows. Data on SARS-CoV-2 viral RNA detection is shown in boxes. The number of daily new cases (with total accumulated cases in brackets) at each sampling date is shown. RTqPCR quantification results obtained with the RdRP reaction are shown. The time point at which lockdown measures were introduced is indicated in red. Source for COVID-19 cases data: https://coronavirus.data.gov.uk/, accessed on 4 July 2020.

Five different RNA replicates from each wastewater concentrate were initially used to generate nPCR products with the different primer combinations shown in Figure S1. As shown in Table 1, positive RT-PCR products were obtained for all five nPCR reactions using RNA extracted from March, April, and May wastewater concentrates. The February wastewater concentrate only produced positive results with nPCR4 and nPCR5 reactions, and only after an additional concentration step to the standard 20–60× concentration procedure was performed (Table 1). The results obtained with nPCR reactions were in good agreement with those from RTqPCR assays as the proportion of positive nPCR reactions closely matched that of the viral RNA concentration values. nPCR assays allowed confirmation by Sanger sequencing and NGS analysis. For all positive samples, positive nPCR results were obtained for at least two different gene targets and from RNA extracted from at least two different independent wastewater concentration processes. nPCR positive reactions produced clean and clear bands following electrophoresis on agarose gels. None of the nPCR reactions with RNA from the wastewater sample collected on 14th January 2020 and none of the multiple RNA extraction and PCR reaction negative controls produced SARS-CoV-2 nPCR products. The RTqPCR and nPCR results are summarized in Figure 3 in the context of epidemiological data.

### 3.4. Nucleotide Sequence Analysis of nPCR Products from Wastewater Concentrates

**Figure 4:**
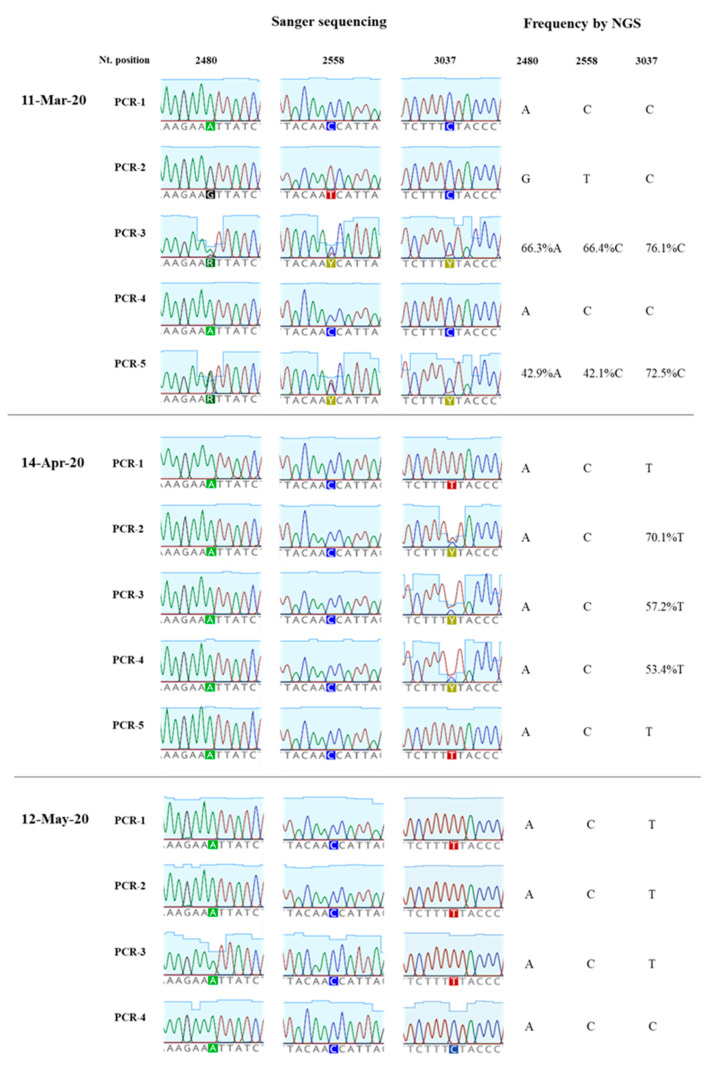
Nucleotide sequence variation in SARS-CoV-2 nPCR1 products from wastewater concentrates. The results of Sanger and NGS analysis of selected nPCR1 products obtained from RNA samples extracted from wastewater concentrates collected on 11th March, 14th April and 12th May are shown.

**Figure 5:**
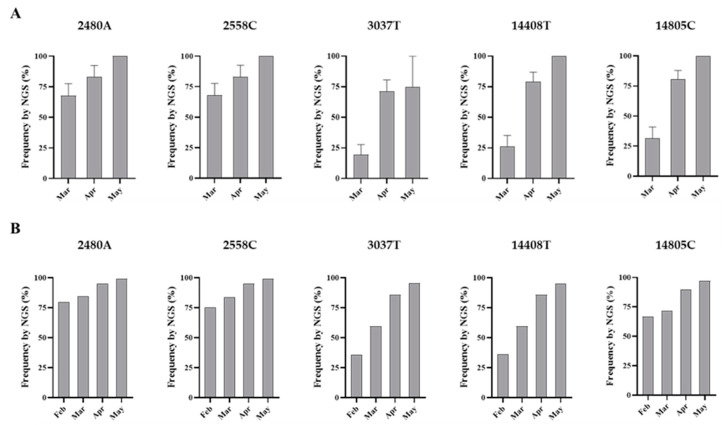
Nucleotide sequence variation at five selected genomic positions in SARS-CoV-2 RNA from wastewater concentrates. Comparison of mean sequence frequency values of nucleotides 2480A, 2558C, 3037T, 14408T, and 14805C found in nPCR1 and nPCR2 products (**A**) are shown and compared with mean sequence frequency values in clinical samples from England for the corresponding months (**B**). Error bars indicate standard error of the mean. Whole-genome SARS-CoV-2 sequences used in this analysis were downloaded from the GISAID database [48].

The amount of SARS-CoV-2 nucleotide sequences obtained by Sanger analysis for each sample is shown in Table 1 and ranged between 847 and 2376 nucleotides per wastewater concentrate. Sequences from several nPCR replicates were generated from each concentrate. Nucleotide sequences for nPCR3, nPCR4, and nPCR5 products for all samples were identical to those of the consensus sequence from clinical samples from England except for few nucleotide changes found in a few nPCR replicates. Nucleotide differences and mixed bases in sequence electropherograms were observed for nPCR1 and nPCR2 products from March and April at nucleotide positions where sequence variations had been observed between clinical samples in England as discussed above. The nPCR products were analysed by NGS with an aim to quantify the proportion of different nucleotides at mixed base positions. Between 82,000 and 200,000 filtered reads were sequenced per nPCR product with >99% of reads typically mapping to SARS-CoV-2 reference sequences. An example of the results for both Sanger and NGS analyses of nPCR1 products obtained with different RNA replicates from each sample are shown in Figure 4. NGS quantification results were in excellent agreement with those observed in Sanger sequence electropherograms although no mixed peaks were detected in Sanger sequences when the minor nucleotide component was below 20%, showing the inferior sensitivity of the Sanger sequence analysis. Differences in sequence composition were found between RNA replicates from samples from March and April reflecting the presence of virus mixtures in both samples, 15 replicate nPCR1 and nPCR2 products were sequenced from each sewage concentrate. Mean sequence frequency values at the five selected nucleotide positions for each month are shown in Figure 5.

Overall, the nucleotide sequence composition at all five selected nucleotide positions changed between March and April. Viral RNA samples containing A2480G and C2558T nucleotide variations decreased between March and April. The proportion of T at positions 3037 and 14,408, genetic markers of the G614 dominant strain, increased between March and April. Finally, the predominant sequence at position 14,805 also switched from T in March to C in April. The same trend in sequence composition continued in May although a similar in-depth analysis was not possible since fewer replicate nPCRs were sequenced successfully. No mixed bases were identified by Sanger or NGS analysis in any of the nPCR products from the RNA samples from May. Sequence results from four nPCR1 replicates from the May sewage found an A at nucleotide 2480 and a C at residue 2558 in all four replicates and a T at position 3037 in 3/4 of the replicates in agreement with their predominance observed in April. A single nPCR2 product sequenced from May also contained the nucleotide sequences of G614 dominant strain at positions 14,408 and 14,805. Few additional sequence variations were identified in few PCR products, but none were present in more than one replicate.

## 4. Discussion

We detected SARS-CoV-2 RNA in wastewater samples collected between February and May 2020. A sample from 14th January was negative and only low levels of viral RNA were detected in the sample from 11th February, 11 days after the first two COVID-19 cases had been confirmed in York, northern England, and 3 days before the first case was reported in the population sampled in our study. The SARS-CoV-2 RNA concentration estimated in wastewater samples was consistent with the number of cases reported at the time of sample collection. Limitations in testing capacity early during the pandemic meant that there was an underestimation of cases in the community, and the extent of community transmission. It is therefore likely that the number of cases in early March was higher, which agrees with the viral RNA levels we found in sewage. Our results showed a large reduction of viral RNA concentration in sewage between April and May, most likely due to the lockdown measures introduced in the country from 23rd March (Figure 3). This is in very good agreement with the observed reduction in COVID-19 confirmed cases and infection estimates [26,45]. However, more frequent sampling would be required to estimate the rate of virus decay and establish firm conclusions about the relationship between cases and what is detected by ES.

Previous studies have detected SARS-CoV-2 RNA in wastewater samples worldwide [9,10,11,12,13,14,15,16,17,18] highlighting ES as a potential tool to help establish early warning systems for the detection of peaks in virus circulation to be able to direct timely public health interventions. The turnaround of laboratory results could take as little as 48 h using our current workflow. However, efforts to improve laboratory methods for sample processing, virus concentration, and viral RNA quantification might be needed to increase the sensitivity for SARS-CoV-2 detection to ensure our ability to detect asymptomatic virus transmission, particularly in areas with low background transmission rates. The type of samples analysed, e.g., raw sewage versus primary sludge or different sample processing e.g., analysing aqueous versus solid phases may have an impact on viral RNA recovery from sewage [12,49,52]. The use of reference standards and collaborative studies between different laboratories using common samples would help in this process, allowing comparability between laboratories and methods. In addition, more detailed mathematical modelling studies similar to those conducted for poliovirus ES [53] will be required to understand the representativeness of replicate sampling, develop sampling strategies around high-risk communities, and establish how ES can best complement clinical diagnosis to hopefully help prevent future lockdowns. Early efforts conducted in Australia [9] to estimate the proportion of individuals infected with SARS-CoV-2 in a catchment area using ES data should be expanded. Although some relevant data are available, more detailed data on the dynamics of SARS-CoV-2 virus excretion in stools are necessary to conduct these analyses, such us knowing the proportion of infected individuals excreting virus in stools, the duration of virus shedding and the virus titres excreted during that period. Estimating the total amount of stool shed per person per day during SARS-CoV-2 infection as well as the virus recovery rate from sewage in the laboratory would be additional factors to increase the accuracy of the modelling.

A novel nested RT-PCR approach targeting five different regions of the viral genome improved the sensitivity of RT-qPCR results. Next generation sequencing analysis of RT-PCR products revealed single nucleotide polymorphisms at five selected nucleotide positions, where sequence variation between viral RNA in clinical samples from England had been observed confirming the co-circulation of virus variants and changes in virus variant predominance with time. The target nucleotide sites for our study were selected following analysis of whole-genome SARS-CoV-2 sequences from the GISAID sequence database [48]. Differences in sequence composition at these positions were notable between different countries/regions during the first few months of the pandemic, but in all cases, SARS-CoV-2 strains containing common nucleotide sequences at these five positions survived, largely reflecting the global dominance of SARS-CoV-2 variant G614 (Figure 2). The G614 variant, containing a glycine (G) at residue 614 in the SARS-CoV-2 spike protein, has become the dominant pandemic form globally showing a consistent increase at all national, regional, and local levels, which suggests a possible fitness advantage [42]. However, no evidence exists yet that any observed changes among SARS-CoV-2 isolates, including G614, have resulted in adaptation to the human host, increased transmissibility, or worsening disease severity [33,34,35,36,37,38,39,40,41]. Other possible explanations for G614 dominance exist such as being caused by purely neutral sampling processes as described for other viruses during previous pandemics [54]. Nucleotide sequences characteristic of variant G614 were present in 36% of the viral RNA sequences reported from England in February but increased to around 60% in March, 86% in April, and 95% in May. Variants A2480G and C2558T were specifically prevalent in clinical samples from England. A combination GT or AT at these two nucleotide positions was present in 8.5% of clinical samples from England but only in 1.1% of clinical samples from the rest of the world, which means that 78.4% of clinical samples showing that combination globally have been reported in England, with that number rising to 92.1% if all U.K. clinical samples are included. The proportion of clinical samples containing variants A2480G and C2558T decreased in England from around 25% in February to 16% in March, 5% in April, and 0.8% in May. Similarly, clinical samples containing variation T14805C dropped from 28.1% in March to 10.4% in April and 2.9% in May (Figure 2). Our sequencing results of SARS-CoV-2 RNA from wastewater samples are consistent with these nucleotide sequence evolution patterns (Figure 5). The nucleotide sequence composition changed at all five selected positions between the samples collected on 11th March and 14th April. Variants A2480G and C2558T were only detected in low proportion in April and 3037T, 14408T, and 14805C became the dominant sequences at these sites consistent with G614 global dominance [42]. In line with our sequence variation results, a study that analysed sequence variation among U.K. SARS-CoV-2 isolates, found that the epidemic comprises a very large number of importations due to inbound international travel [55]. The rate and source of introduction of SARS-CoV-2 lineages into the U.K. changed rapidly through time, peaking in mid-March, with most introductions occurring during March 2020. Many U.K. transmission lineages appeared to be very rare or extinct at the time of reporting (8th June). This would be consistent with notable changes expected in virus population dynamics with a likely decrease in sequence heterogeneity from mid-March onwards as seen in our results from sewage.

Overall, our study shows that ES can be used to detect SARS-CoV-2 transmission with viruses identified in sewage resembling those found in clinical samples. We were able to detect virus variants specifically circulating in England and also to identify changes in viral RNA sequences consistent with the increasing global dominance of G614 pandemic variant [42]. Our results are encouraging and suggest a potentially wider applicability of ES to monitor SARS-CoV-2 transmission, tracing virus variants and detecting virus importations.

## 5. Conclusions

We have shown that environmental surveillance can be used to monitor SARS-CoV-2 transmission detecting virus variants specifically circulating in England and identifying changes in virus variant predominance known to have occurred during the COVID-19 pandemic. Environmental surveillance could be used for the early detection of peaks in virus transmission for public health interventions to be timely implemented.

## Figures and Tables

**Table 1:** Detection of Severe Acute Respiratory Syndrome Coronavirus 2 (SARS-CoV-2) viral RNA in wastewater concentrates by RTqPCR and nPCR.

	SARS-CoV-2 RTqPCR (No. of Replicates) ^1^		Nested RT-PCR Gene Target (PCR Product Size) ^2^					Genome Sequenced (No. of Nucleotides)
Sampling Date	*RdRP Gene* (log_10_ gc/L Sewage)	*E Gene* (log_10_ gc/L Sewage)	*nsp2-PLPro* *Gene* nPCR1 (714 nt)	*RdRP* *Gene* nPCR2 (523 nt)	*RdRP* *Gene* nPCR3 (527 nt)	*RdRP* *Gene* nPCR4 (235 nt)	*ORF8b-N* *Gene* nPCR5 (612 nt)	
14-Jan-20	-	-	-	-	-	-	-	-
11-Feb-20	-	-	-	-	-	+	+	847
11-Mar-20	4.84 ± 0.45 [4.18–5.52] (*n* = 10)	4.98 ± 0.40 [4.63–5.41] (*n* = 3)	+	+	+	+	+	2376
14-Apr-20	5.27 ± 0.30 [4.77–5.91] (*n* = 11)	5.78 ± 0.07 [5.71–5.84] (*n* = 3)	+	+	+	+	+	2376
12-May-20	<3.5 (*n* = 11) ^3^	-	+	+	+	+	+	2376

Wastewater samples were concentrated using a standard filtration–centrifugation method (concentration factor: 20–60×).^1^ Mean values of log10 SARS-CoV-2 genome copy (SC2 gc)/L wastewater with standard deviations are shown. ^2^ Dark grey indicates positive in at least 1/5 replicate nPCR reactions. Light grey indicates positive only after additional concentration (up to 500×). Positive PCR results were obtained for Feb–May samples in at least two independent concentration processes for at least two different gene targets. The January sample remained negative even after a second concentration step.^3^ Only 3/11 replicates gave positive RTqPCR signals with RdRP target, so viral RNA quantification was not possible.

## Supplementary Materials

The following are available online at https://www.mdpi.com/1999-4915/12/10/1144/s1, Figure S1: Nested-PCR strategy. Table S1: Number of whole-genome SARS-CoV-2 sequences by region/country and date of collection analysed in this study. Table S2: Most common nucleotide sequence combinations at five selected nucleotide sites in SARS-CoV-2 clinical samples. Tables S3–S9 include acknowledgments for authors who submitted the sequences analysed.
