# Supplementary material for: Tracking SARS-CoV-2 in Sewage: Evidence of Changes in Virus Variant Predominance during COVID-19 Pandemic
Source: Viruses. 2020 Oct 9;12(10):1144. doi: 10.3390/v12101144 (PMC7601348; doi:10.3390/v12101144)
Supplement: Supplementary file 1 [file viruses-12-01144-s001.zip › Figure S1.pdf]

SARS-CoV-2 genome

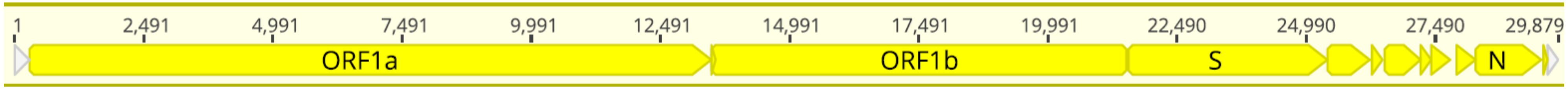

NGS1F + NGS1R

NGS2F + NGS2R ***nPCR1 (714 nt)***

NGS3F + NGS3R

NGS4F + NGS4R ***nPCR2 (523 nt)***

PCR1F + PCR1R

PCR2F + PCR2R ***nPCR3 (527 nt)***

PCR3F + PCR3R ***nPCR4 (235 nt)***

PCR5F + PCR5R

PCR6F + PCR6R ***nPCR5 (612 nt)***

Primer Name	Genome nt. Position	Primer Sequence (5'-3')
COVID-NGS1F	2267-2293	GCAAAGGAAATTAAGGAGAGTGTTCAG
COVID-NGS1R	3243-3265	AGTTGTCTGATTGTCCTCACTGC
COVID-NGS2F	2344-2368	CATTATTGGTGGAGCTAAACTTAAAGCC
COVID-NGS2R	3091-3118	AGTACCATACTCATATTGAGTTGATGGC
COVID-NGS3F	14266-14292	ACGGAAGAGAGGTTAAAACTCTTTGAC
COVID-NGS3R	15112-15139	TCATAGTACTACAGATAGAGACACCAGC
COVID-NGS4F	14342-14365	GTTTGGATGACAGATGCATTCTGC
COVID-NGS4R	14889-14913	GTTAGCATTAATACAGCCACCATCG
COVID-PCR1F	14909-14932	CTAACCAAGTCATCGTCAACAACC
COVID-PCR1R	16110-16134	GTGTCCTGTTAACTCATCATGTAGC
COVID-PCR2F	15285-15312	GGGTTGGGATTATCCTAAATGTGATAGA
COVID-PCR2R	15840-15865	GTCCTTTAGTAAGGTCAGTCTCAGTC
COVID-PCR3F	15488-15509	GAGATGCCACAACTGCTTATGC
COVID-PCR3R	15745-15769	CCACTAGACCTTGAGATGCATAAGT
COVID-PCR5F	27917-27942	AATCATCACAACTGTAGCTGCATTTC
COVID-PCR5R	28831-28853	AACTGTTGCGACTACGTGATGAG
COVID-PCR6F	28099-28123	CACCCATTCAGTACATCGATATCGG
COVID-PCR6R	28736-28757	GAAGTTGTAGCACGATTGCAGC
