# Supplementary material for: Tracking SARS-CoV-2 in Sewage: Evidence of Changes in Virus Variant Predominance during COVID-19 Pandemic
Source: Viruses. 2020 Oct 9;12(10):1144. doi: 10.3390/v12101144 (PMC7601348; doi:10.3390/v12101144)
Supplement: Supplementary file 1 [file viruses-12-01144-s001.zip › Table_S1.pdf]

Table S1. Number of whole-genome SARS-CoV-2 sequences by region/country and date of collection analysed in this study

Date	England	Spain	Asia	USA
**Up to Feb-20**	114	17	1047	122
**Mar-20**	6359	1399	1437	5737
**Apr-20**	9244	354	1094	3980
**May-20**	2364	36	618	1032
**Total**	18081	1806	4196	10871
