# Supplementary material for: Tracking SARS-CoV-2 in Sewage: Evidence of Changes in Virus Variant Predominance during COVID-19 Pandemic
Source: Viruses. 2020 Oct 9;12(10):1144. doi: 10.3390/v12101144 (PMC7601348; doi:10.3390/v12101144)
Supplement: Supplementary file 1 [file viruses-12-01144-s001.zip › Table_S2.pdf]

TABLE S2. Most common nucleotide sequence combinations at 5 selected nucleotide sites in SARS-CoV-2 clinical samples

Variant	2480	2558	3037	14408	14805
**1**	A	C	C	C	C
**2**	A	C	C	C	T
**3**	G	T	C	C	T
**4**	A	T	C	C	T
**5**	A	C	T	T	C
